# The Use and Abuse of Pessaries

**Published:** 1883-06

**Authors:** J. Matthews Duncan


### THE USE AND ABUSE OF PESSARIES.

PROFESSOR J. MATTHEWS DUNCAN, M. D., LL. D.

I feel that I do not myself know the straight, scientific road to the clean and sweet drops of truth I would like to present. Consequently I shall deal much in negatives; not this way nor that is the right one. Now, in the present great abundance of contorted bits of wood, and metal, and vulcanite, and what not, called pessaries, my advice is: Think twice before beginning the often baneful practice of using any instrument. Many a woman has suffered from, many a woman has died of, a pessary; but most pessaries are nearly inocuous for evil or for good. They are always harborers of dirt, keep the mind watching the part, liable to decay, and are undesirable additions to the contents of the pelvic excavation, and, if they are efficient, must, of course, cause more pressure—though perhaps on different parts—than that caused by the organ or organs which they keep in an altered position. You sometimes see what are called galvanic pessaries, whose object is to act otherwise than mechanically, giving a homœopathic dose of galvanism. These pessaries are used in amenorrhœa and in virgins, and to all this kind of meddling there are strong objections, medical and moral. Look upon pessaries as a surgeon looks on a truss, not medicinal otherwise than as a mechanical means of procuring healing, comfort, and safety to your patient.

Of virgins, I may say that there is very rarely occasion to examine for displacement, and examination can generally be done quite satisfactorily per rectum. You get the knowledge of the condition of the pelvic viscera that you want, and that is all you should require. If you find only a minor displacement, you had better let it alone, not even trying a pessary. It is only in very rare, complicated cases with distinct mechanical indications that a pessary should be tried or used. I do not remember using one on any ground whatever in a virgin.

Intra-uterine or stem pessaries are the only instruments you can rely on for straightening the uterus or keeping a flexion undone. They do this as a male bougie straightens the urethra. Some kinds have an outside or pubic part by which the straightened uterus is fixed; but the oldest kind and the most recent respect the mobility of the uterus. They have been three times introduced into practice within this century, but the practice has never flourished. Many modifications have been ingeniously devised with a view to perfect them, but in vain. They are far more injurious and dangerous than the conditions they are intended to modify.

The evils of intra-uterine pessaries have led to great ingenuity in attempts to undo flexions and keep them undone by vaginal—not intra-uterine instruments. This attempt is often successful in retroflexion which does not occur as a congenital rigidly fixed condition, and can be dealt with just as a retroversion is managed. But the curious things are anteflexion pessaries, and in regard to their giving relief, I meantime express no opinion ; but I do say that if they give relief it is not by undoing the flexion and keeping it undone—keeping the womb straight. I have seen most kinds of anteflexion pessaries as placed by their inventors, and too often replaced and replaced, but I have never seen one materially modify the flexion. I have myself never used one, and have no intention of doing so. There is another bad and too common practice, which I must not omit to mention here : that is, what is called straightening or putting up the womb or replacing it time after time by the probe or finger. This has no other effect than to irritate the organ, for the displacement recurs immediately after the probe or finger is removed, as the practice itself shows.

It is not a simple matter to judge of the part taken by a pessary in relieving or removing painful symptoms. A kindly doctor makes an amiable patient anxious to please him and ready to express a sense of relief which may not be real. Besides, you will find many patients alarmed at the idea of having a displacement, and, believing the pessary undoes it or cures it, wear an instrument with satisfaction and even pleasure, although it gives them new pains or increases what they had before. In such difficulties, how are you to be guided ? The difficulty is almost insuperable if your patient has become possessed by erroneous notions of the importance of displacements, and you must take care to prevent the adoption of such notions.

What do you expect from a pessary? You may replace a descended or retroflexed or retroverted uterus and keep it replaced by a pessary, and you may so relieve or remove pains. You cannot cure a displacement, though sometimes you can substitute one displacement for another: that is, for example, change a retroversion into an anteversion. No doubt, a displacement may sometimes be, in a sense, cured—as when an adhesive perimetritis ends in tying a uterus up to the higher part of the sacrum. But all kinds of minor displacements are incurable by any kind of instrumental treatment. Remove the instrument, and the displacement is just as it was before, or there is a new alternative one, and this however long the instrument may have been in place.

Displacements sometimes disappear. Thus a woman with chronic inflammation of the cervix, and probably also relaxation of the vagina, gets rid of these conditions and then the uterus ascends from its descended, and perhaps flexed, position. A woman with a bulky uterus—perhaps containing a small fibroid—becomes aged; the uterus becomes lighter and lighter, and the upper vagina contracts and the descended uterus ascends. Any change in the constitution of the abdomen which increases its retentive power will raise the uterus higher, destroying displacement, and such changes in the abdomen may result from enlargement of the base of the thorax, or from changes in the quantity and disposition of fat.

I have already said that a pessary often cures by its effect on the mind. A patient recently said to me: "You have quite cured me. I can walk now; but not without that pessary." And she was not altogether pleased when I told her she had no pessary—that I had removed it months previously without her being aware of my having done so. I had omitted to tell her. Had she known she had no pessary she would have found pains arise from walking, and all this without any desire to be untrue.

A pessary often gives relief, even when small, and having no discoverable function—doing nothing. Of the occasional occurrence of such cases I do not doubt, and I am quite unable to explain them. It is of such cases I was thinking when I told you that practical success must overrule theory, or take the place of a failure in theory. It is quite common to find a pessary give relief in what may be called a flexion, because that feature of the case is most striking, without the pessary changing the flexion. In such cases the pessary may maintain a diminished degree of descent, and may prevent increase of descent on walking and may save a tender part of the uterus from pressure on sitting. There is no difficulty in explaining such cases; but to comprehend the action of the pessary you should think of the case as one of descent, not of flexion, and this is true of almost all—if not all—cases of flexion.

As a matter of fact, I find the majority of versions and flexions—as observed in practice and treated by pessaries—have their whole conditions of displacement quite unaltered by the pessary, even while in.

One of the best examples of relief by the pessary is observed in the anteversion (by probe) of an engorged retroverted and descended uterus. Here a well-fitted Hodge is comforting and curative, maintaining the anteversion, elevating the uterus, or preventing descent on walking or standing, and preventing relapse into retroversion or retroflexion by keeping the posterior laquear of the vagina pressed against the sacrum.

Another notable example of relief is seen in descent with tendency to cystocele, when the irritation of the cystocele pushing at the orifice of the vagina is most annoying. In such, a suitably sized Hodge, or India-rubber ring often, by its anterior limb, just catches the cystocele and obviates the tendency to protrusion through the os vaginæ.

For each case your pessary must be specially adapted: a flat, a boat-shaped, or a double-curved, and it must fit the patient in size and contour. Nothing can instruct you in this but bedside experience. Occasionally you have to try more pessaries than one to find the most suitable. Sometimes a woman whose case you expected to relieve by pessary can bear none—of whatever kind.

A pessary, if it is to be useful, will give relief at once, and will need very little attention from you. If you are frequently fitting and re-adapting, you are almost surely doing more harm than good. A well-fitted pessary may be worn for months without being attended to. You must take care that the pessary does not cause ulceration and cut the vagina, and you must have a new one placed when the former one gets decayed.

You will find it hard to get any good from a pessary unless you have a fair amount of perineum to support it. A pessary will be inefficient if the vagina is not long enough and capacious enough to allow of its action without strong pressure on the vaginal wall.

In flexion or version without descent of the whole organ you can do no good to the version or flexion by a pessary; you have no basis or fulcrum to work from.—*Medical Times and Gazette*, Dec. 30th, 1882.

---

Niagara Useful at Last.—We have it from good authority that the American Institute meets this year at Niagara Falls, in order that the roar of the cataract may drown the nonsense of their debates. So Niagara *has* its uses after all!!

Query: If a drop of Aconite were put in Niagara River at Buffalo, what "Fluxion Centesimal" potency would it be at Lewiston?

